# Calculated versus measured iron losses and instantaneous magnetization power functions of electrical steel

**DOI:** 10.1007/s00202-021-01474-4

**Published:** 2022-01-25

**Authors:** Helmut Pfützner, Georgi Shilyashki, Emanuel Huber

**Affiliations:** 1 Institute of EMCE, TU Wien, 1040 Vienna, Austria; 2 ViennaMagnetics GmbH, 1020 Vienna, Austria

**Keywords:** Iron losses, Eddy currents, Hysteresis, Instantaneous magnetization power, Loss testers

## Abstract

Magnetic energy loss *P* of soft magnetic laminations like SiFe sheets tends to be expressed through an integral over the power product *H* · d*B*/d*t*. Already in earlier papers, we stressed that distinctions are needed for the quantities *H* and *B*. However, they are not considered in practically consistent ways, in the so far literature. Here, we discuss these distinctions in closer ways, comparing loss determination by calculation and measurement, respectively. A physically consistent procedure is described for the determination of loss and magnetization power functions through measurement of bi-located quantities *H*_S_ and *B*_C_ (S surface, C cross section). On the other hand, it is concluded that corresponding quantitative calculations—based on co-located quantities *H* and *B*—are impeded by the high amount of technological parameters of modern steel products. For example, they result from chemical additions, and—in particular—also from specific technologies of rolling, annealing, coating or scribing.

## Introduction

Soft magnetic laminations like silicon iron sheets represent the standard material for cores of electric machines. Since hundred years, intensive attempts are made to reduce the magnetic energy loss of core materials, supported by standards for their experimental testing [1, 2]. Traditionally, the loss is determined for the so-called technical frequencies of grid systems, i.e. for *f* = 50 Hz or 60 Hz. However, during the last years, higher frequencies, up to an order of several kHz, gained industrial relevance. This is due to widened applications for electric drives, in particular for electro-mobility.

Magnetic loss is expressed by a quantity *P* that usually is given in W/kg, i.e. related to the mass of material. For example, grain-oriented (GO) steels as applied for power transformers show very low values *P* < 1 W/kg for a peak induction value *B*_PEAK_ = 1.7 T. This is significant with respect to long-term operation. On the other hand, in the case of electric drives, loss values up to the order of 100 W/kg are accepted, however, mainly for non-oriented (NO) steel that is loaded for a rather short duration of time with *f* > 50 Hz.

Historically, the above means that the practically relevant range of loss *P* has been strongly extended, from less than 1 W/kg, up to 100 W/kg. As a consequence, accurate loss determination gains industrial significance. This is valid for both, the experimental measurement and the mathematical calculation. In both cases, it is a convention to express loss quantities in a time-averaged way by a function of the following type:

1 $$ 1/\left( {\rho T} \right) \cdot _{\,{T}} \!\!\!\int {H(t)} \cdot {\text{d}}B/{\text{d}}t \cdot {\text{d}}t. $$

Here, *ρ* is the density of sample. *T* = 1/*f* is the length of the period of sinusoidal magnetization with frequency *f*. *B* stands for induction, *H* for magnetic field strength.

As a reason of confusion, the literature uses different definitions for the two quantities *B* and *H*, partly also replacing *B* through the—practically identical—polarization *J*. Different text books on magnetisms tend to use different definitions, partly lacking the corresponding physical conditions.

In a surprising way, this is even valid for international standards. In particular, IEC standards [1] for Epstein Tester and [2] for Single Sheet Tester (SST) replace the induction *B* by the polarization *J* (in T), without any closer definition or comment. The quantity *H* is introduced as the “magnetic field strength”, as well without further specification. For the first time, in 1978, Pfützner [3] proved that two different definitions can be chosen, in order to determine either mere hysteresis loss or total loss.

## Physical preconditions

As it is well known, expressions of type (1) are derived from Maxwell Equations (MEs). For magnetic problems, the latter offer a link between the vector field quantities magnetic field strength ***H***, the induction ***B***, the electric field strength ***E*** and the current density ***S*** = *γ* ***E*** (with *γ* the conductivity). A further interdependency is given by the vacuum permeability (magnetic constant) *µ*_o_, according to ***B*** = *µ*_o_ ***H*** + ***J***. All above quantities are defined for a location of the soft magnetic matter, as a macroscopic continuum. The level below is described by the Lorentz-Maxwell equations [4], as a link between microscopic mechanisms and macroscopic ones.

As a characteristic of a heterogeneously built-up soft magnetic steel sample, it offers itself to define five levels (Table 1), in rather unsystematic ways, due to overlaps. The steel consists of crystals that may show diameters up to many mm, in particular in the case of GO SiFe. For example, talking about “induction ***B***”, we usually address the induction that results from an average over several grains and introduce this quantity into the system of MEs. Analytically or numerically, we finally can determine the “local” distribution of induction, also as a global quantity, e.g. averaged over a given region of the magnetic object.

**Table 1:** Here distinguished (overlapping) levels of modelling and analysis

		Calculation tools	Experimental tools
L5	Global (or regional)	MEs/FEM	Sensors
L4	Local	MEs/FEM	
L3	Macroscopic (crystalline)	Lorentz-MEs	Microscopy
L2	Microscopic	Domain theory	Optic, X-ray
L1	Sub-microscopic	Quantum/thermodyn modelling	

As a specific problem for the here discussed case, calculated field quantities like ***B*** and ***H*** correspond to each other, for a given “co-location” and a given point of time. For example, they express induction and field in the inner centre of a sample—or at any other location, i.e. volume element d*V*, respectively. On the other hand, comparable experimental measurements are not possible in co-local ways, due to the lack of sufficiently small sensors. Moreover, almost not any sensor types are given for interior measurements.

Apart from exceptional indirect methods, as described in [5], measurements of the local induction ***B*** in an individual grain of GO-steel on Level L3 use smallest search coils in holes that are drilled into the tested lamination. In a—usually much smaller—grain of NO-steel, a corresponding measurement cannot be performed at all. A search coil tends to yield an average over several grains, corresponding to L5. The corresponding vectors of field ***H*** can hardly be measured at all. Smallest tangential field coils or Hall plates show dimensions of many mm for effective tests. Thus, they yield results on level L5, a priori.

For the here given task of comparing calculated loss determinations with experimental one, the above conditions cause problems of restricted comparability. Analytical or numerical calculations tend to yield local results on L4 that can be handled by Maxwell equations (MEs). Of course, they can be averaged for the determination of regional results. On the other hand, experiments are restricted to regional results, a priori. This means that their results cannot be handled through MEs in straightforward ways.

In clear ways, it follows from MEs that mere hysteresis losses at a given local volume element d*V* can be expressed as

2 $$ P_{{\text{H}}} = {1}/\left( {\rho T} \right) \cdot _{\,{\text{T}}}\!\! \int {H\left( t \right)} \cdot {\text{d}}B/{\text{d}}t \cdot {\text{d}}t. $$

Here, *H* is the magnetic field at the given location d*V*, *B* is the corresponding local induction. On the other hand, for the first time, it was proved in [3] that for an infinitely long cylindric body of arbitrary cross section *A*, total averaged losses can be expressed by

3 $$ P \, = {1}/\left( {\rho T} \right) \cdot _{\,{\text{T}}}\!\! \int {H_{{\text{S}}} \left( t \right)} \cdot {\text{d}}B_{{\text{C}}} /{\text{d}}t \cdot {\text{d}}t = { 1}/T \cdot _{\,{\text{T}}}\!\! \int {p\left( t \right)} {\text{ d}}t . $$

Under the condition of axial excitation, *H*_S_ is the magnetic field as measured at the surface, averaged over some distance that covers a sufficiently large amount of grains. *B*_C_ is the induction averaged over the cross section *A*. *p*(*t*) is the magnetization power function.

As already mentioned above, even standards [1, 2] do not distinguish between the above two cases (2) and (3). But this is also valid for the literature, including major text books, according to the following examples:

- Bozorth [6], p. 508 expresses *B*(*H*) locally, for mere *P*_H_,
- Kneller [7], p. 612 *B*(*H*) locally, for mere *P*_E_,
- Bertotti [8], Ch. 12 *B*(*H*_a_) locally, for loss portions, with *H*_a_ as the applied field,
- Handley [9], p. 339 *B*(*H*), for total *P*,
- Fiorillo [10] p. 367 J(*H*_a_) for cross section and surface, respectively, for measurement of total *P*,
- Coey [11] (p. 442) *B*(*H*), for total *P,*
- Hilzinger [12] (p. 121) *B*(*H*), for total *P*,
- Moses [13] (p. 167) *B*(*H*_a_) for cross section and surface, respectively, for measurement of total *P*.

Not any of the above references offers a distinction between Eqs. (2) and (3). This causes errors of interpretation in scientific studies, but even more in industrial discussions.

As a resulting problem, also the term “hysteresis loop” is used in ambivalent ways. Per definition, it is applied for the quasi-static case where the loop area expresses mere hysteresis loss. But the literature uses also the term “dynamic hysteresis loop”. In fact, due to a very weak change of hysteresis with varying frequency *f*, this loop is very weakly affected by varying *f*. A well-known mechanism is the nucleation of main Bloch walls with rising *f*, as being typical for highly GO steel [14, 15]. However, for the following, we neglect small differences between quasi-static and dynamic hysteresis loops.

On the other hand, in accordance with [3], we distinguish between “hysteresis loops” and “dynamic magnetization loops” that are characterized by a distinct widening for increasing frequency *f*. For electric steel, the main reason for this widening is that a dynamic loop is not determined by a measurement at a single defined locality, i.e. in a *co-local* way, according to level L4. In all usual practice, it is measured in a *bi-local* way (on level L5), *H*_S_ at the surface of sample and *B*_C_ as an average over its cross section.

For some plausibility of the mechanism of widening, let us consider the external field *H*_S_ at the surface (as location 1) as the driving source of progressing external and internal induction *B* through Bloch wall displacements. The initiated time-changes of *B* cause eddy currents that affect the inner magnetization (as location 2) in two ways: (i) reducing its intensity, and (ii) causing a lag of phase (see, e.g. [6], p. 770]). Both mechanisms are most pronounced in the sheet centre, but in weaker ways also for the average of cross section, i.e. for *B*_C_. The overall process involves two mechanisms of dissipative nature—hysteresis heating through wall movements, and eddy current heating through electric currents. This corresponds to the sum of hysteresis loss and eddy current loss, in full accordance with the analytical treatment of [3].

We assume that performing a (fictive) co-local measurement of both *H*_S_ and *B*_S_ at the surface, the loop function *B*_S_(*H*_S_) would hardly be affected by increased frequency *f*. A thought experiment would result in a very similar loop *B*_C_(*H*_C_), from a co-local measurement that is taken as an average over the cross section. On the other hand, the practically usual, bi-local measurement of *H*_S_ and *B*_C_ reveals a widened dynamic loop, the area of which represents the sum of hysteresis loss and eddy current loss.

For the practical design of loss testers, we addressed the needed distinctions in several publications like [16–18]. Here, we discuss them through a systematic comparison of calculation and measurement of magnetic loss *P* and instantaneous magnetization power functions *p*(*t*).

## Experimental loss measurement

According to international standards, loss *P* is measured either by the already mentioned Epstein Tester [1] or by the Single Sheet Tester [2]. In the latter case, the major part of sample is magnetized in almost homogeneous ways as sketched in Fig. 2.

**Fig. 1:**
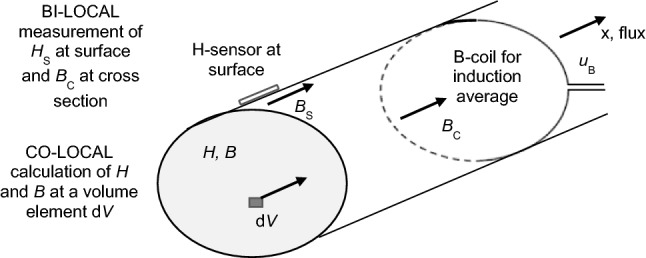
Soft magnetic cylinder of infinite length, magnetized in axial direction *x*, with a surface field strength *H*_S_. Calculations yield co-local values *H* and *B* for a given volume element d*V*. Measurements yield bi-local values *H*_S_ for the surface by means of a field sensor, and *B*_C_ as averaged over the cross section, by means of a search coil winding, via the induced voltage *u*_B_

**Fig. 2:**
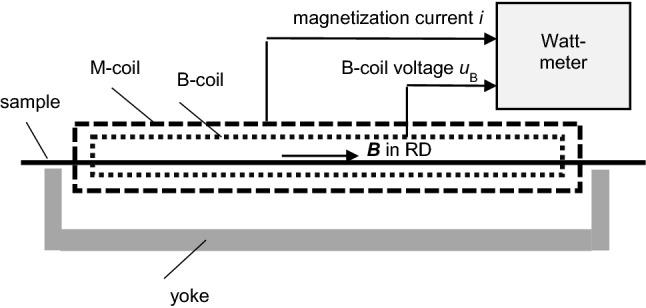
Sample of a soft magnetic lamination, magnetized in rolling direction (RD) by a so-called Single Sheet Tester (SST). According to IEC standards, the induction *B*—according to polarization *J*—is detected by an induction coil (B-coil). The magnetic field strength *H* is indirectly determined from the magnetization current *i*. The loss *P* is derived by the Watt-metric principle

For loss measurements, the induction tends to be detected by a B-coil round the sample. As an alternative, it can be detected through the so-called needle method. In both cases, the result reflects the induction *B*_C_ that is averaged over the samples cross section. Actually, it is not possible to measure the induction *B*_S_ of the sample surface, approximate determinations being restricted to evaluations of surface positions of main domain Bloch walls.

According to standardization, the magnetic field strength is determined indirectly by a measurement of the magnetization current *i*. As a drawback, the result of measurement is affected by the yoke system, in particular also by the contact regions between sample ends and yoke. A more effective alternative is the arrangement of a tangential field coil (H-coil) on the sample surface (e.g. [16–18]). As an option, a tangential Hall plate is applied (e.g. [16, 19]). All three cases yield the magnetic field strength *H*_S_ of the sample surface. Measurements of interior field values *H* are not possible, apart from approximate X-ray testing.

The physically clearest measurement of loss *P* is performed by the well-known rise-of-temperate method. However, in cases of low *P*, it yields poor resolution. Thus, it tends to be replaced by the so-called electro-dynamic principle.

**Fig. 3:**
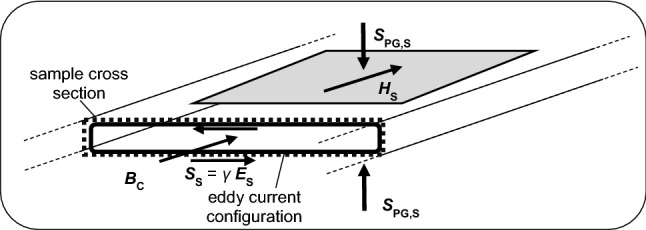
Central, quasi-homogeneously magnetized detection region of a 500 mm × 500 mm sized sample of thickness *d*, magnetized in RD by a novel Low Mass Single Sheet Tester. According to Poynting theorem, losses are determined from the magnetic surface field *H*_S_ as detected by a tangential H-coil of 300 mm × 480 mm size. The electric surface field *E*_S_ in transverse direction (TD) is determined from the voltage of a (not shown) B-coil that covers the 300 mm long region, in consistent ways. Magnetization is performed by a (not shown) surrounding M-coil of 430 mm length

Figure 3 sketches an alternative to the standardized SST, as realized by our recently developed “Low Mass SST” for measurements at IEC-standardized samples of 500 mm × 500 mm size. Striving for physical consistency, the tester is characterized by the arrangement of a B-coil and an H-coil in the same (quasi-homogeneous) sample region, averaged over the rather large area of 300 mm length and 480 mm width. With the further above, this yields the cross section-averaged induction *B*_C_ and the surface field *H*_S_.

As already mentioned, in 1978, reference [3] proved in analytical ways for an arbitrary cross section that the latter two quantities *B*_C_ and *H*_S_ allow a determination of loss *P* in exact ways. However, a condition is that *H*_S_ is a constant along the whole surface. Later, in 2018, we confirmed this in [18] for the more simple case of a plane lamination of constant thickness *d*, as it is illustrated by Fig. 3.

As a basis, we apply the surfaces Poynting vector ***S***_PG,S_ [3, 8, 10, 13, 17, 18]. In Fig. 3, it is assumed directed upwards below the sheet and downwards above it, with identical intensities

4 $$ S_{{{\text{PG}},{\text{S}}}} = \, \left| {{\varvec{E}}_{{\text{S}}} \times {\varvec{H}}_{{\text{S}}} } \right| \, = E_{{\text{S}}} \cdot H_{{\text{S}}} . $$

Here, *S*_PG,S_ represents the instantaneous power density that “streams” into the material, related to a surface area element. The aim of our measurement is to determine the total loss *P* averaged over both the duration *T* of the period and the sheets cross section and also related to the materials density *ρ.* Due to both-sides power stream, the loss results as

5 $$ P \, = {2 }/ \, \left( {T\rho d} \right) _{\,\,\,{\text{T}}}\! \int {H_{{\text{S}}} \left( t \right)} \cdot E_{{\text{S}}} \left( t \right) \cdot {\text{d}}t. $$

It is assumed to be quasi-constant along both length in rolling direction (RD) and width in transverse direction (TD), corresponding to values that are well averaged over the structure of grains and their domains.

As a very effective option, we measure *H*_S_(*t*) by means of a tangential field coil (H-coil) of just 2 mm thickness. For good averaging, its length is close to 300 mm, the width is almost 500 mm. With an effective distance of little more than 1 mm from the sample surface, the first ME promises that the detected field equals the samples surface field *H*_S_ in very good approximation. It follows from the coils voltage *u*_H_ prop. d*H*_S_/d*t*, through numerical integration.

The second to be detected quantity is the surfaces electric field *E*_S_(*t*). Easily, it can be determined from the voltage *u*_B_ of a B-coil of 300 mm length around the sample. The second ME yields the simple relation

6 $$ {\text{d}}B_{{\text{C}}} /{\text{d}}t = { 2}E_{{\text{S}}} /d, $$

proportional to the B-coil voltage *u*_B_.

Finally, the above procedure yields the total loss *P*, related to the materials density *ρ*, according to (3).

Unfortunately, so far, no golden standard exists for the measurement of *P*. However, the above described procedure is characterized by a high degree of physical consistency, thus promising good absolute accuracy. Residual sources of systematic error result from the effective distance of H-coil from the sample surface and from the problem that the B-coil windings have some distance from the sample surfaces. Both mechanisms are considered by careful manufacturing of hardware, supported by some software correction (by field extrapolation, and by air flux compensation, respectively). With these measures, the accuracy can be estimated as being close to about 2%, i.e. distinctly better than that of standardized measurements on the basis of Watt-metric measurement of total power consumption that includes also loss of the magnetization yoke system.

At this point, we would like to add a thought experiment. According to the above, total losses are measurable in bi-local ways, i.e. linking surface field *H*_S_ with cross section averaged induction *B*_C_. Further, we stressed that local measurements of surface *B*_S_ or field *H* at an interior location are not possible in practice. Assuming that we produce very small sensors for co-local measurements of induction *B* and field *H* at any single point of sample, then the experiment would yield mere hysteresis loss *P*_H_, according to the further down Eq. (9). Assuming that we also produce a very small sensor for the measurement of the current density *S* at this location, the equation would allow for the additional determination of the local eddy current loss *P*_E_. The sum would offer the *local* total loss *P*.

## Calculation of losses

As it is well known, the Maxwell equations (MEs) describe vector interdependencies of the electric field strength ***E***, the dielectric polarization ***D***, the current density ***S***, the magnetic field strength ***H*** and the induction ***B***. Let us now consider an electromagnetically excited volume element of solid matter. For it, the MEs yield in well-known ways the instantaneous, local electromagnetic power density as

7 $$ p_{{{\text{EM}},{\text{L}}}} (t) \, = { 1}/\gamma {\varvec{S}}^{2} \, + {\varvec{H}}\,{\text{d}}{\varvec{B}}/{\text{dt }} + {\varvec{E}}\,{\text{d}}{\varvec{D}}/{\text{d}}t. $$

The first term is the Ohmic power density, the current density ***S*** being assumed to represent mere eddy currents, i.e. no galvanically impressed currents. The second term is the magnetization power density, the third one is the electric polarization power density that is neglected in the following.

Averaged over the sample thickness, we find

8 $$ p_{{{\text{EM}}}} (t) \, = { 1}/d _{{\text{d}}} \int {\left[ {{1}/\gamma S^{2} + H{\text{d}}B/{\text{d}}t} \right]} {\text{ d}}z. $$

**Fig. 4:**
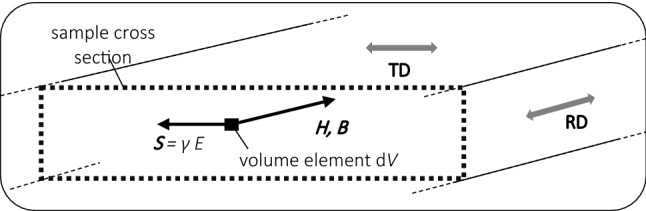
Soft magnetic lamination, magnetized in RD. For an inner volume element d*V*, the field vector ***H***, the induction vector ***B*** and the current density vector ***S*** are indicated, as they have to be calculated in analytical or numerical ways

Let us now consider a soft magnetic sheet of thickness *d* as sketched in Fig. 4. We assume that it is magnetized in the rolling direction where ***H*** and ***B*** are parallel to each other, without other components. The crucial task is to perform an effective analytical or numerical calculation of local quantities ***S***, ***H*** and ***B*** that are needed in order to determine the instantaneous thickness-averaged (or volume-averaged) power density.

Theoretically, the latter promises a calculation of the total loss *P*. Per definition, it is related to the density *ρ* of material and averaged over the duration *T* of the period. With (3) and (8), this yields

$$ \begin{aligned} P & = {1}/\left( {\rho \cdot T} \right) _{\,\,{\text{T}}}\! \int {H_{{\text{S}}} \cdot {\text{d}}B_{{\text{C}}} /{\text{d}}t \cdot {\text{d}}t} \\ & = {1}/\left( {d \cdot \rho \cdot T} \right)_{\,\,{\text{T}}}\! \int {_{d} \int { \, \left[ {{ 1}/\gamma \cdot S^{2} \, + H \cdot {\text{d}}B/{\text{d}}t} \right]} } {\text{ d}}z \cdot {\text{d}}t \\ \end{aligned} $$

The equation indicates two possible strategies for the calculation of total losses *P*:

(1) Straightforward analytical or numerical calculation of the two key quantities *H*_S_(*t*) and *B*_C_(*t*)—a task that hardly is possible in practice.

(2) Analytical or numerical calculation of the quantities *H*(*t*), *B*(*t*) and *S*(*t*), for all locations of the sheet, followed by averaging over the cross section. This would yield eddy current loss as

10 $$ P_{{\text{E}}} = {1}/\left( {d \cdot \rho \cdot T} \right) _{\,\,{\text{T}}}\! \int {_{d} \int {{1}/\gamma \cdot S^{2} } } {\text{ d}}z \cdot {\text{d}}t. $$

Provided that the calculation of the eddy current configuration ***S*** resolves the 3D structure as resulting from both the classical and the excess currents round moving Bloch walls, Eq. (10) in vector expression would reflect the sum of classical and excess eddy current losses—but this is not feasible, in practice due to technological conditions as discussed further down.

In analogous ways, we find hysteresis loss as

11 $$ P_{{\text{H}}} = {1}/\left( {d \cdot \rho \cdot T} \right) _{\,\,{\text{T}}}\! \int {_{d} \int { \, \left[ {H \cdot {\text{d}}B/{\text{d}}t} \right]} } {\text{ d}}z \cdot {\text{d}}t. $$

While the first term of the second line of (9) represents the eddy current loss *P*_E_, the second term represents the mere hysteresis loss *P*_H_ [3], contrary to frequent misinterpretations in the literature. In particular, technically oriented literature like [12] does not distinguish between cross section-averaged field quantities and surface quantities, as a source of error.

Unfortunately, the textbooks define the field quantity in demanding ways. In particular, several authors introduce *H*_a_ as the “applied” field which however may differ from the samples surface field *H*_S_ in strong ways through effects of demagnetization. Further, dynamic “hysteresis” loops are introduced as functions *B*(*H*) of strong frequency dependence. This is in contrast to the already mentioned fact that hysteresis loss *P*_H_ depends on frequency in very weak ways. As already hint to in Sect. 2 and in accordance with [3], we suggest the following, clearly distinguishing definitions:

“Dynamic hysteresis loop”—as the local function *B*(*H*) that describes for a given volume element d*V* the very weak dependence of mere hysteresis from frequency *f*,

“Dynamic magnetization loop”—as the global function *B*_C_(*H*_S_) that comprises both hysteresis and eddy currents, thus depending on *f* in strongest ways.

The literature reports many attempts to calculate individual portions of loss (e.g. [8], Ch. 12], [9], Ch. 9.4], [10], Ch. 2], [13], Ch.], [13], Ch. 7], [20, 21]). Most effective results are attained for “classical” eddy current loss. As, e.g. described in [7] Ch. 40] or [8] Ch. 12], it neglects the existence of domain structures. But even with this significant simplification, the material’s nonlinearity restricts the results to approximations. Hysteresis loss depends on many parameters that impede effective calculations, a priori. Further, the literature postulates the so-called excess losses, as a third portion that is a crucial challenge for calculation, as pointed out in [13], Ch. 7.8]. In contrast, measurement of losses on the basis of (3) promises to yield total losses *P* that represent the main demand for industrial applications—irrespective of physically relevant portions.

Anyhow, apart from a priori problematic tools of calculation, it should be considered that the performance of modern steel products tends to be steadily increased by company-specific technological measures. The latter include seemingly minor changes of chemical composition (through so-called additions), conditions of rolling and annealing, as well as after-treatments like stress coating or laser scribing. Even best tools of calculation are not likely to consider the corresponding consequences on quantities like *S*, *E*, *H* and *B*, the knowledge of which would be needed for mathematical loss estimations.

As a conclusion, we assume that mathematical loss predictions are not feasible, apart from rough predictions within a given class of steel, based on parameter fitting. As a benefit, loss separation favours discussions of frequency dependencies, as also stressed in [8], Ch. 12.4.3]. They also allow for simulations of possible consequences of technological modifications of a given product of steel. However, in a general way, for practice, loss calculation does not represent an effective alternative for experimental measurements.

## Measured instantaneous magnetization power functions

According to (3), for the definition of instantaneous magnetization power functions, we relate to the density, according to

10 $$ p\left( t \right) \, = { 1}/\rho \cdot H_{{\text{S}}} \left( t \right) \cdot {\text{d}}B_{{\text{C}}} /{\text{d}}t. $$

This yields a quantity in W/kg, thus offering direct comparisons with the time-averaged total loss *P*.

First attempts for corresponding calculations by Petrun et al. [22, 23] were focused on portions of power, i.e. hysteresis and eddy current power, in virtually sliced sheets. However, calculations are complicated by all problems that were expressed in Sect. 3 with respect to loss *P*. Thus, we restrict the following to the experimental determination.

Recently, we reported in [24] our first measured power function *p*(*t*) for grain-oriented steel, demonstrating that instantaneous values *p* may offer effective conclusions on the temporal “history” of loss generation. For the measurement, we applied the Low Mass SST according to Fig. 2. Its B-coil voltage *u*_B_ yields d*B*_C_/d*t* in a direct way. The H-coil voltage *u*_H_(*t*) yields *H*_S_(*t*) after numerical integration.

**Fig. 5:**
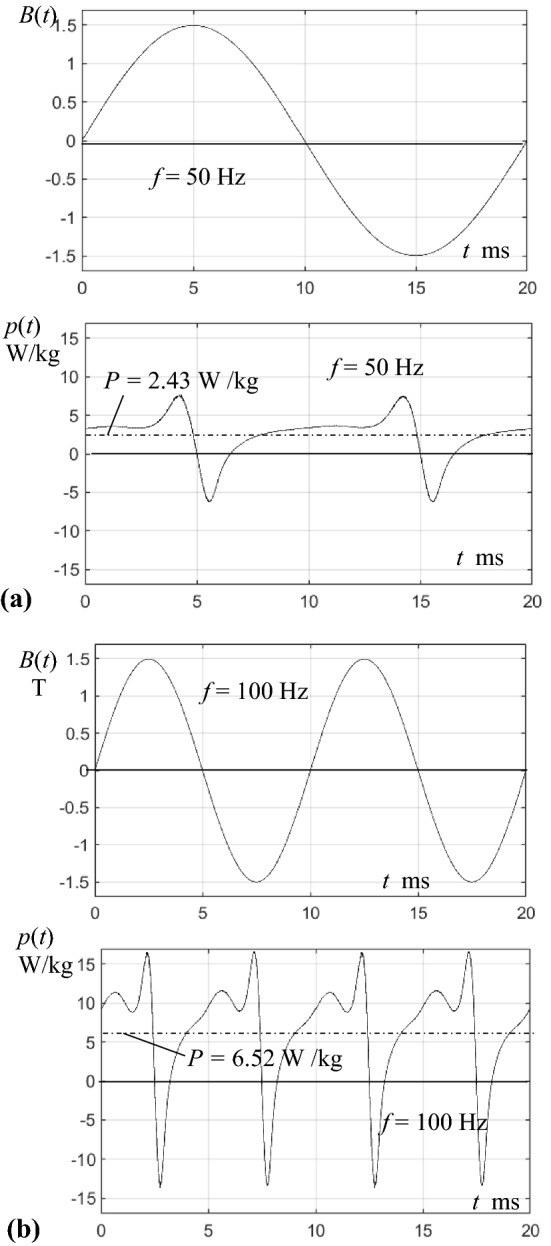
Instantaneous power functions *p*(*t*), for a sample of NO-steel, magnetized with a peak induction *B*_C,PEAK_ = 1.5 T. **a** One period for a frequency *f* = 50 Hz. **b** Two periods for *f* = 100 Hz (with same scaling)

While the just mentioned function *p*(*t*) in [24] concerned a GO steel type, Fig. 5 depicts first functions for a 500 µm thick non-oriented (NO) steel type of time-averaged loss *P* = 2.43 W/kg for 50 Hz and *B*_PEAK_ = 1.5 T. Figure 5a shows *p*(*t*) for sinusoidal induction. Close to the instant *t* = 2 ms, *p* exhibits a peak value *p*_PEAK_ = 3 W/kg, that indicates that peak losses may exceed averaged ones in distinct ways. Round *t* = 5 ms and 15 ms, we see a pair of spikes that are due to turns of atomic moments, thus being of non-dissipative nature.

Figure 5b shows corresponding results for doubled frequency *f* = 100 Hz, where averaged loss results as *P* = 6.52 W/kg, corresponding to an increase of 170%. On the other hand, *p*(*t*) reveals an instantaneous peak loss, according to about *p*_PEAK_ = 12 W/kg. Also, the intensity of non-dissipative spikes is strongly increased, the negative spike showing − 13 W/kg, instead of − 6 W/kg for 50 Hz.

As a conclusion, instantaneous power functions can be measured in easy ways. They yield both practically and theoretically relevant information about the “history” of temporal development, as arising during a period of magnetization.

## Discussion and conclusions

As early as in 1978, the first author of the present paper pointed to the finding that the literature on magnetic power loss *P* uses different interpretations of terms of type _o_∫ *H* · d*B* (see Eq. (1)). Reference [3] expressed the need to define values *H* and *B* as specific local values of field and induction, respectively, for the determination of mere hysteresis loss *P*_H_. On the other hand, for total loss *P*, bi-locally determined values *H*_S_ and *B*_C_ were suggested for the surface and for an average over the materials cross section, respectively. The regional difference is characterized by phase shifts through eddy currents, as a main reason for different results.

So far, the above distinction has not found common use. Thus, the present paper discusses practical aspects and consequences of lacking distinction. In particular, the latter is a possible explanation for too low values of calculated losses. On the other hand, measured losses are less problematic, since they tend to be based on bi-locally quantities *H*_S_ and *B*_C_, a priori.

For the here also addressed instantaneous power functions *p*(*t*), the suggested relation to density shows the advantage that both *P* and *p* are given in W/kg, which favours interpretations. On the other hand, the distinction between dissipative and non-dissipative portions needs deeper theoretical knowledge. Unfortunately, a restricted distinction yields errors of interpretation that may be of high practical significance.

## References

[1] IEC Standards 60404-2 (1996) Methods of measurement of the magnetic properties of magnetic sheet and strip by means of an Epstein tester

[2] IEC Standards 60404-3 (1992) Methods of measurement of the magnetic properties of magnetic sheet and strip by means of a single sheet tester

[3] Pfützner H (1978). Zur Bestimmung der Ummagnetisierungsverluste aus den Feldgrößen. Arch Elt.

[4] Lorentz HA (1937) Versuch einer Theorie der elektrischen und optischen Erscheinungen in bewegten Körpern. Springer Coll. Pap. 1–138

[5] Pfützner H, Shilyashki G (2015). Magnetic dummy sensors—a novel concept for interior flux distribution tests. Int J Appl Electrom Mech.

[6] Bozorth RM (1951). Ferromagnetism.

[7] Kneller E (1962). Ferromagnetismus.

[8] Bertotti G (1998). Hysteresis in magnetism.

[9] O’Handley RC (2000). Modern magnetic materials.

[10] Fiorillo F (2004). Measurement and characterization of magnetic materials.

[11] Coey JMD (2010). Magnetism and magnetic materials.

[12] Hilzinger R, Rodewald W (2013). Magnetic materials.

[13] Moses A, Anderson P, Stanbury H (2019) Electrical steels: I. Fundamentals and basic concepts. IET

[14] Pry RH, Bean CP (1958). Calculation of the energy loss in magnetic sheet materials using a domain model. J Appl Phys.

[15] Pfützner H, Bishop S, Harasko G (1994). A multi-parametric domain model for highly grain oriented polycrystalline Si-Fe. J Magn Magn Mater.

[16] Pfützner H (1980). Investigation of silicon iron by means of Hall probes and stochastic ergodic correlation. J Magn Magn Mater.

[17] Pfützner H, Schönhuber P (1991). On the problem of the field detection for single sheet testers. IEEE Trans Magn.

[18] Pfützner H, Shilyashki G (2018). Theoretical basis for physically correct measurement and interpretation of magnetic energy losses. IEEE Trans Magn.

[19] Perevertow O (2005). Measurement of the surface field on open magnetic samples by the extrapolation method. Rev Sci Instrum.

[20] Landgraf FJG, de Campos MF, Leicht J (2008). Hysteresis loss subdivision. J Magn Magn Mater.

[21] Pluta WA (2010). Measurements of magnetic properties of electrical steel sheets for the aim of loss separation. J Electr Eng.

[22] Petrun M, Podlogar V, Steentjes S, Hameyer K, Dolinar D (2014). IEEE Trans Magn.

[23] Petrun M, Steentjes S (2020). Iron-loss and magnetization dynamics in non-oriented electrical steel: 1-D excitations up to high frequencies. IEEE Access.

[24] Pfützner H, Shilyashki G, Huber E (2020). Physical assessment of the magnetic path length of energy loss testers. IEEE Trans Magn.

